# *catena*-Poly[[diaqua­dibromidoman­ganese(III)]-μ-pyridine-2-carboxyl­ato]

**DOI:** 10.1107/S160053680902844X

**Published:** 2009-07-25

**Authors:** Nam-Ho Kim, Kwang Ha

**Affiliations:** a School of Applied Chemical Engineering, the Research Institute of Catalysis, Chonnam National University, Gwangju 500-757, Republic of Korea

## Abstract

The asymmetric unit of the title compound, [MnBr_2_(C_6_H_4_NO_2_)(H_2_O)_2_]_*n*_, contains one monomeric unit of the neutral linear coordination polymer. The Mn^3+^ ions are bridged by anionic pyridine-2-carboxyl­ate (pic) ligands, thereby forming a chain-like structure along the *c* axis, and are six-coordinated in a distorted octa­hedral environment by two O atoms of the two different carboxyl­ate groups, two O atoms of two water mol­ecules and two Br atoms. The complex displays inter­molecular O—H⋯Br, O—H⋯N, O—H⋯O, C—H⋯O and C—H⋯Br hydrogen bonding. There may also be inter­molecular π–π inter­actions between adjacent pyridine rings, with a centroid–centroid distance of 3.993 (8) Å.

## Related literature

For the synthesis and structure of [Mn(pic)_3_], see: Figgis *et al.* (1978 ▶); Yamaguchi & Sawyer (1985 ▶); Li *et al.* (2000 ▶). For the synthesis and structure of [Mn(pic)_2_(H_2_O)_2_], see: Okabe & Koizumi (1998 ▶); Barandika *et al.* (1999 ▶). For details of mono-, di- and polynuclear Mn(II, III, IV)–pic complexes, see: Huang *et al.* (2004 ▶). For the synthesis and structure of the anionic Mn(II)–pic polymer, {[MnBr_2_(pic)(H_2_O)]^−^}_*n*_, see: Kim *et al.* (2009 ▶).
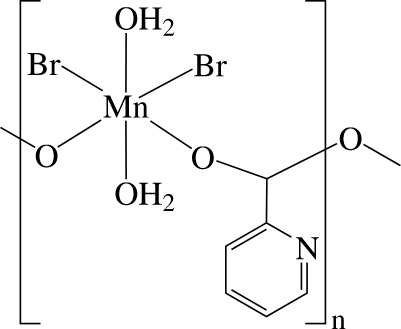


## Experimental

### 

#### Crystal data

[MnBr_2_(C_6_H_4_NO_2_)(H_2_O)_2_]
*M* *_r_* = 372.89
Monoclinic, 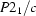

*a* = 10.290 (3) Å
*b* = 13.814 (4) Å
*c* = 7.978 (3) Å
β = 109.810 (6)°
*V* = 1066.9 (6) Å^3^
*Z* = 4
Mo *K*α radiation
μ = 8.71 mm^−1^
*T* = 223 K
0.25 × 0.23 × 0.10 mm

#### Data collection

Bruker SMART 1000 CCD diffractometer
Absorption correction: multi-scan (*SADABS*; Bruker, 2000 ▶) *T*_min_ = 0.133, *T*_max_ = 0.418
6572 measured reflections
2168 independent reflections
1510 reflections with *I* > 2σ(*I*)
*R*_int_ = 0.060

#### Refinement

*R*[*F* ^2^ > 2σ(*F* ^2^)] = 0.070
*wR*(*F* ^2^) = 0.248
*S* = 1.14
2168 reflections
127 parameters
H-atom parameters constrained
Δρ_max_ = 2.85 e Å^−3^
Δρ_min_ = −1.46 e Å^−3^

### 

Data collection: *SMART* (Bruker, 2000 ▶); cell refinement: *SAINT* (Bruker, 2000 ▶); data reduction: *SAINT*; program(s) used to solve structure: *SHELXS97* (Sheldrick, 2008 ▶); program(s) used to refine structure: *SHELXL97* (Sheldrick, 2008 ▶); molecular graphics: *ORTEP-3* (Farrugia, 1997 ▶) and *PLATON* (Spek, 2009 ▶); software used to prepare material for publication: *SHELXL97*.

## Supplementary Material

Crystal structure: contains datablocks global, I. DOI: 10.1107/S160053680902844X/im2127sup1.cif

Structure factors: contains datablocks I. DOI: 10.1107/S160053680902844X/im2127Isup2.hkl

Additional supplementary materials:  crystallographic information; 3D view; checkCIF report

## Figures and Tables

**Table 1:** Hydrogen-bond geometry (Å, °)

*D*—H⋯*A*	*D*—H	H⋯*A*	*D*⋯*A*	*D*—H⋯*A*
O3—H3*A*⋯Br1^i^	0.83	2.58	3.340 (9)	154
O3—H3*B*⋯N1^ii^	1.10	2.41	3.466 (14)	162
O4—H4*A*⋯Br2^iii^	0.83	2.70	3.333 (9)	135
O4—H4*A*⋯O1^iii^	0.83	2.33	2.908 (14)	127
O4—H4*B*⋯Br1^iv^	1.02	2.31	3.210 (9)	147
C2—H2⋯O4^v^	0.94	2.59	3.319 (18)	134
C4—H4⋯Br2^vi^	0.94	2.80	3.534 (12)	135

Symmetry codes: (i) 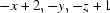; (ii) 
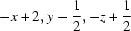
; (iii) 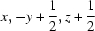; (iv) 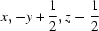; (v) 
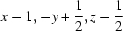
; (vi) 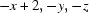.

**Fig. 1.**
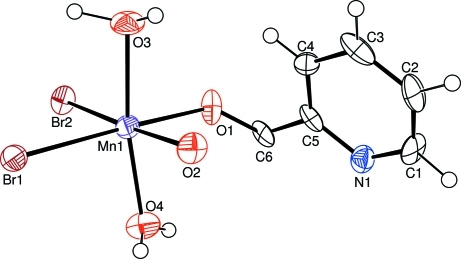
The repeat unit of the title compound, with displacement ellipsoids drawn at the 50% probability level for non-H atoms.

**Fig. 2.**
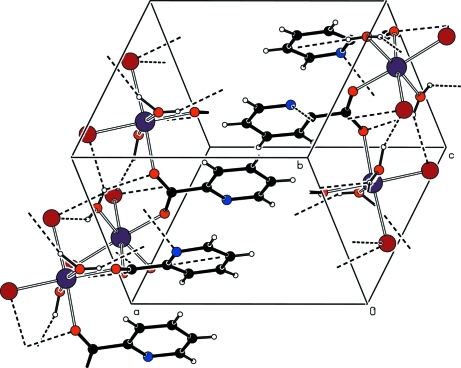
View of the unit-cell contents and chain-like structure of the title compound. Hydrogen-bond interactions are drawn with dashed lines.

## supplementary crystallographic information

### Comment

Coordination polymers are attracting great attention because of their potential applications such as in catalysis, magnetism, molecular recognition and other fields (Huang *et al.*, 2004).

The asymmetric unit of the title compound, [MnBr_2_(C_6_H_4_NO_2_)(H_2_O)_2_]_n_, contains one monomeric unit of the neutral linear coordination polymer (Fig. 1). Mn^3+^ ions are bridged by anionic pyridinecarboxylate (pic) ligands, thereby forming a one-dimensional zigzag chain-like structure along the *c* axis (Fig. 2). Mn^3+^ ions are six-coordinated in a distorted octahedral environment by two O atoms of the two different carboxylate groups, two O atoms of two water molecules and two Br atoms. Water molecules are *trans* with respect to each other, whereas Br atoms and O atoms of the carboxylate groups are *cis* with respect to each other, respectively. The complex displays intermolecular O—H···Br, O—H···N, O—H···O, C—H···O and C—H···Br hydrogen bonding (Table 1 and Fig. 2). There may also be intermolecular π-π interactions between adjacent pyridine rings, with a centroid-centroid distance of 3.993 (8) Å. The structure of the complex polymer is comparable with the structure of the anionic complex polymer, {[MnBr_2_(pic)(H_2_O)]^-^}_n_, in which the Mn^2+^ ions are linked to each other by pyridinecarboxylate bridges in a *syn*-anti mode (Kim *et al.*, 2009).

### Experimental

A solution of MnBr_2_ × 4 H_2_O (0.920 g, 3.208 mmol) and pyridine-2-carboxylic acid (0.200 g, 1.625 mmol) in H_2_O (10 ml) was refluxed for 3 h. The solvent was removed in vacuum, the residue was dissolved in MeOH/H_2_O (5 ml/5 ml) and filtered. After evaporation of the solvent, the residue was dried at 333 K, to give a pale pink powder (0.918 g). Crystals suitable for X-ray analysis were obtained by slow evaporation from a CH_3_CN solution.

### Refinement

H atoms were positioned geometrically and allowed to ride on their respective parent atoms [C—H = 0.94 Å and *U*_iso_(H) = 1.2*U*_eq_(C)]. The H atoms of the water molecules were located from Fourier difference maps, but not refined [*U*_iso_(H) = 1.5*U*_eq_(O)].

### Crystal data

**Table:**

[MnBr_2_(C_6_H_4_NO_2_)(H_2_O)_2_]	*F*(000) = 712
*M**_r_* = 372.89	*D*_x_ = 2.321 Mg m^−^^3^
Monoclinic, *P*2_1_/*c*	Mo *K*α radiation, λ = 0.71073 Å
Hall symbol: -P 2ybc	Cell parameters from 2889 reflections
*a* = 10.290 (3) Å	θ = 2.6–28.2°
*b* = 13.814 (4) Å	µ = 8.71 mm^−^^1^
*c* = 7.978 (3) Å	*T* = 223 K
β = 109.810 (6)°	Plate, colorless
*V* = 1066.9 (6) Å^3^	0.25 × 0.23 × 0.10 mm
*Z* = 4	

### Data collection

**Table:**

Bruker SMART 1000 CCD diffractometer	2168 independent reflections
Radiation source: fine-focus sealed tube	1510 reflections with *I* > 2σ(*I*)
graphite	*R*_int_ = 0.060
φ and ω scans	θ_max_ = 26.4°, θ_min_ = 2.1°
Absorption correction: multi-scan (*SADABS*; Bruker, 2000)	*h* = −12→11
*T*_min_ = 0.133, *T*_max_ = 0.418	*k* = −17→17
6572 measured reflections	*l* = −5→9

### Refinement

**Table:**

Refinement on *F*^2^	Primary atom site location: structure-invariant direct methods
Least-squares matrix: full	Secondary atom site location: difference Fourier map
*R*[*F*^2^ > 2σ(*F*^2^)] = 0.070	Hydrogen site location: inferred from neighbouring sites
*wR*(*F*^2^) = 0.248	H-atom parameters constrained
*S* = 1.14	*w* = 1/[σ^2^(*F*_o_^2^) + (0.135*P*)^2^ + 7.437*P*] where *P* = (*F*_o_^2^ + 2*F*_c_^2^)/3
2168 reflections	(Δ/σ)_max_ < 0.001
127 parameters	Δρ_max_ = 2.85 e Å^−^^3^
0 restraints	Δρ_min_ = −1.46 e Å^−^^3^

### Special details

**Table:**

Geometry. All e.s.d.'s (except the e.s.d. in the dihedral angle between two l.s. planes) are estimated using the full covariance matrix. The cell e.s.d.'s are taken into account individually in the estimation of e.s.d.'s in distances, angles and torsion angles; correlations between e.s.d.'s in cell parameters are only used when they are defined by crystal symmetry. An approximate (isotropic) treatment of cell e.s.d.'s is used for estimating e.s.d.'s involving l.s. planes.
Refinement. Refinement of *F*^2^ against ALL reflections. The weighted *R*-factor *wR* and goodness of fit *S* are based on *F*^2^, conventional *R*-factors *R* are based on *F*, with *F* set to zero for negative *F*^2^. The threshold expression of *F*^2^ > σ(*F*^2^) is used only for calculating *R*-factors(gt) *etc*. and is not relevant to the choice of reflections for refinement. *R*-factors based on *F*^2^ are statistically about twice as large as those based on *F*, and *R*- factors based on ALL data will be even larger.

### Fractional atomic coordinates and isotropic or equivalent isotropic displacement parameters (Å^2^)

**Table:**

	*x*	*y*	*z*	*U*_iso_*/*U*_eq_	
Mn1	1.11555 (19)	0.12215 (13)	0.3085 (2)	0.0262 (5)	
Br1	1.27174 (14)	0.06169 (8)	0.63672 (16)	0.0287 (4)	
Br2	1.29649 (14)	0.06032 (9)	0.17035 (17)	0.0322 (4)	
O1	0.9974 (9)	0.1781 (7)	0.0294 (12)	0.037 (2)	
O2	0.9538 (9)	0.1716 (6)	0.4164 (12)	0.033 (2)	
O3	1.0017 (9)	−0.0140 (6)	0.2414 (12)	0.039 (2)	
H3A	0.9255	−0.0079	0.2534	0.059*	
H3B	1.0868	−0.0659	0.2907	0.059*	
O4	1.1968 (11)	0.2673 (6)	0.3608 (12)	0.039 (2)	
H4A	1.1943	0.2857	0.4586	0.058*	
H4B	1.1898	0.3074	0.2510	0.058*	
N1	0.7263 (11)	0.3399 (7)	0.0142 (14)	0.031 (2)	
C1	0.6010 (14)	0.3448 (9)	0.0408 (19)	0.035 (3)	
H1	0.5611	0.4054	0.0464	0.042*	
C2	0.5345 (14)	0.2620 (11)	0.059 (2)	0.041 (4)	
H2	0.4469	0.2652	0.0714	0.049*	
C3	0.5976 (14)	0.1726 (12)	0.0593 (18)	0.042 (4)	
H3	0.5538	0.1153	0.0750	0.050*	
C4	0.7258 (12)	0.1683 (9)	0.0361 (16)	0.027 (3)	
H4	0.7690	0.1083	0.0367	0.032*	
C5	0.7886 (12)	0.2528 (8)	0.0123 (14)	0.025 (3)	
C6	0.9277 (13)	0.2547 (9)	−0.0176 (15)	0.027 (3)	

### Atomic displacement parameters (Å^2^)

**Table:**

	*U*^11^	*U*^22^	*U*^33^	*U*^12^	*U*^13^	*U*^23^
Mn1	0.0264 (10)	0.0225 (9)	0.0289 (10)	−0.0013 (7)	0.0085 (8)	0.0024 (7)
Br1	0.0340 (8)	0.0267 (7)	0.0244 (7)	0.0015 (5)	0.0085 (5)	0.0002 (4)
Br2	0.0366 (8)	0.0356 (8)	0.0276 (7)	0.0081 (5)	0.0149 (6)	0.0039 (5)
O1	0.027 (5)	0.048 (5)	0.036 (5)	0.013 (4)	0.012 (4)	0.016 (4)
O2	0.029 (5)	0.039 (5)	0.038 (5)	0.003 (4)	0.024 (4)	−0.005 (4)
O3	0.042 (6)	0.026 (5)	0.049 (6)	−0.015 (4)	0.015 (5)	0.003 (4)
O4	0.058 (7)	0.033 (5)	0.030 (5)	−0.007 (4)	0.020 (5)	0.000 (4)
N1	0.032 (6)	0.031 (6)	0.028 (6)	0.006 (5)	0.007 (5)	0.003 (4)
C1	0.028 (7)	0.035 (7)	0.053 (8)	0.010 (5)	0.030 (6)	−0.007 (6)
C2	0.015 (6)	0.061 (10)	0.049 (9)	0.005 (6)	0.014 (6)	0.002 (7)
C3	0.024 (7)	0.063 (9)	0.037 (8)	−0.020 (7)	0.008 (6)	−0.002 (7)
C4	0.025 (6)	0.022 (6)	0.033 (7)	−0.004 (5)	0.009 (5)	0.001 (5)
C5	0.020 (6)	0.044 (7)	0.004 (5)	−0.003 (5)	−0.003 (4)	0.001 (4)
C6	0.025 (6)	0.040 (7)	0.008 (5)	−0.003 (5)	−0.005 (4)	0.007 (5)

### Geometric parameters (Å, °)

**Table:**

Mn1—O4	2.158 (9)	N1—C5	1.365 (15)
Mn1—O3	2.184 (8)	N1—C1	1.378 (16)
Mn1—O2	2.224 (8)	C1—C2	1.366 (19)
Mn1—O1	2.281 (9)	C1—H1	0.94
Mn1—Br2	2.608 (2)	C2—C3	1.40 (2)
Mn1—Br1	2.699 (2)	C2—H2	0.94
O1—C6	1.261 (14)	C3—C4	1.394 (18)
O2—C6^i^	1.217 (14)	C3—H3	0.94
O3—H3A	0.83	C4—C5	1.378 (16)
O3—H3B	1.10	C4—H4	0.94
O4—H4A	0.83	C5—C6	1.529 (18)
O4—H4B	1.02	C6—O2^ii^	1.217 (14)
			
O4—Mn1—O3	171.1 (4)	Mn1—O4—H4B	115
O4—Mn1—O2	86.1 (4)	H4A—O4—H4B	129
O3—Mn1—O2	87.1 (3)	C5—N1—C1	120.9 (11)
O4—Mn1—O1	85.2 (4)	C2—C1—N1	120.3 (12)
O3—Mn1—O1	89.3 (3)	C2—C1—H1	119.9
O2—Mn1—O1	93.0 (3)	N1—C1—H1	119.9
O4—Mn1—Br2	95.8 (3)	C1—C2—C3	119.5 (12)
O3—Mn1—Br2	90.8 (3)	C1—C2—H2	120.3
O2—Mn1—Br2	177.4 (3)	C3—C2—H2	120.3
O1—Mn1—Br2	85.3 (2)	C4—C3—C2	119.9 (13)
O4—Mn1—Br1	92.1 (3)	C4—C3—H3	120.1
O3—Mn1—Br1	93.7 (2)	C2—C3—H3	120.1
O2—Mn1—Br1	89.9 (2)	C5—C4—C3	119.5 (12)
O1—Mn1—Br1	175.9 (2)	C5—C4—H4	120.3
Br2—Mn1—Br1	91.89 (7)	C3—C4—H4	120.3
C6—O1—Mn1	129.5 (8)	N1—C5—C4	120.0 (12)
C6^i^—O2—Mn1	136.7 (9)	N1—C5—C6	117.1 (10)
Mn1—O3—H3A	110	C4—C5—C6	122.9 (11)
Mn1—O3—H3B	100	O2^ii^—C6—O1	130.0 (13)
H3A—O3—H3B	134	O2^ii^—C6—C5	115.9 (11)
Mn1—O4—H4A	109	O1—C6—C5	114.1 (10)
			
O4—Mn1—O1—C6	−47.0 (11)	C2—C3—C4—C5	−0.3 (19)
O3—Mn1—O1—C6	125.9 (11)	C1—N1—C5—C4	0.4 (17)
O2—Mn1—O1—C6	38.8 (11)	C1—N1—C5—C6	−179.8 (10)
Br2—Mn1—O1—C6	−143.2 (11)	C3—C4—C5—N1	1.0 (18)
O4—Mn1—O2—C6^i^	−3.1 (12)	C3—C4—C5—C6	−178.8 (10)
O3—Mn1—O2—C6^i^	−177.3 (12)	Mn1—O1—C6—O2^ii^	107.2 (14)
O1—Mn1—O2—C6^i^	−88.1 (12)	Mn1—O1—C6—C5	−73.5 (12)
Br1—Mn1—O2—C6^i^	89.0 (12)	N1—C5—C6—O2^ii^	−19.0 (15)
C5—N1—C1—C2	−2(2)	C4—C5—C6—O2^ii^	160.8 (12)
N1—C1—C2—C3	3(2)	N1—C5—C6—O1	161.6 (10)
C1—C2—C3—C4	−2(2)	C4—C5—C6—O1	−18.6 (15)

Symmetry codes: (i) *x*, −*y*+1/2, *z*+1/2; (ii) *x*, −*y*+1/2, *z*−1/2.

### Hydrogen-bond geometry (Å, °)

**Table:**

*D*—H···*A*	*D*—H	H···*A*	*D*···*A*	*D*—H···*A*
O3—H3A···Br1^iii^	0.83	2.58	3.340 (9)	154
O3—H3B···N1^iv^	1.10	2.41	3.466 (14)	162
O4—H4A···Br2^i^	0.83	2.70	3.333 (9)	135
O4—H4A···O1^i^	0.83	2.33	2.908 (14)	127
O4—H4B···Br1^ii^	1.02	2.31	3.210 (9)	147
C2—H2···O4^v^	0.94	2.59	3.319 (18)	134
C4—H4···Br2^vi^	0.94	2.80	3.534 (12)	135

Symmetry codes: (iii) −*x*+2, −*y*, −*z*+1; (iv) −*x*+2, *y*−1/2, −*z*+1/2; (i) *x*, −*y*+1/2, *z*+1/2; (ii) *x*, −*y*+1/2, *z*−1/2; (v) *x*−1, −*y*+1/2, *z*−1/2; (vi) −*x*+2, −*y*, −*z*.
